# Targeted oligonucleotide-mediated microsatellite identification (TOMMI) from large-insert library clones

**DOI:** 10.1186/1471-2156-6-54

**Published:** 2005-11-15

**Authors:** Kefei Chen, Christoph Knorr, Kirsten Bornemann-Kolatzki, Jun Ren, Lusheng Huang, Gary A Rohrer, Bertram Brenig

**Affiliations:** 1 Institute of Veterinary Medicine, University of Göttingen, Burckhardtweg 2, 37077, Göttingen, Germany; 2 Key Laboratory for Animal Biotechnology of Jiangxi Province and the Ministry of Agriculture, Jiangxi Agricultural University, Nanchang 330045, P. R. China; 3 US Meat Animal Research Center, Agricultural Research Service, US Department of Agriculture, Clay Center, NE 68933-0166, USA

## Abstract

**Background:**

In the last few years, microsatellites have become the most popular molecular marker system and have intensively been applied in genome mapping, biodiversity and phylogeny studies of livestock. Compared to single nucleotide polymorphism (SNP) as another popular marker system, microsatellites reveal obvious advantages. They are multi-allelic, possibly more polymorphic and cheaper to genotype. Calculations showed that a multi-allelic marker system always has more power to detect Linkage Disequilibrium (LD) than does a di-allelic marker system [1]. Traditional isolation methods using partial genomic libraries are time-consuming and cost-intensive. In order to directly generate microsatellites from large-insert libraries a sequencing approach with repeat-containing oligonucleotides is introduced.

**Results:**

Seventeen porcine microsatellite markers were isolated from eleven PAC clones by **t**argeted **o**ligonucleotide-**m**ediated **m**icrosatellite **i**dentification (TOMMI), an improved efficient and rapid flanking sequence-based approach for the isolation of STS-markers. With the application of TOMMI, an average of 1.55 (CA/GT) microsatellites per PAC clone was identified. The number of alleles, allele size distribution, polymorphism information content (PIC), average heterozygosity (H_T_), and effective allele number (N_E_) for the STS-markers were calculated using a sampling of 336 unrelated animals representing fifteen pig breeds (nine European and six Chinese breeds). Sixteen of the microsatellite markers proved to be polymorphic (2 to 22 alleles) in this heterogeneous sampling. Most of the publicly available (porcine) microsatellite amplicons range from approximately 80 bp to 200 bp. Here, we attempted to utilize as much sequence information as possible to develop STS-markers with larger amplicons. Indeed, fourteen of the seventeen STS-marker amplicons have minimal allele sizes of at least 200 bp. Thus, most of the generated STS-markers can easily be integrated into multilocus assays covering a broader separation spectrum. Linkage mapping results of the markers indicate their potential immediate use in QTL studies to further dissect trait associated chromosomal regions.

**Conclusion:**

The sequencing strategy described in this study provides a targeted, inexpensive and fast method to develop microsatellites from large-insert libraries. It is well suited to generate polymorphic markers for selected chromosomal regions, contigs of overlapping clones and yields sufficient high quality sequence data to develop amplicons greater than 250 bases.

## Background

Almost all of the applied protocols to isolate microsatellites de novo include construction of partial genomic libraries (selected for small insert size) followed by cumbersome screening steps with hybridization probes [2]. Here, we introduce an improved approach called TOMMI (**T**argeted **O**ligonucleotide-**M**ediated **M**icrosatellite **I**dentification) to develop microsatellites by straightforward sequencing of clones isolated from large-insert libraries like PAC (**P**1-derived **A**rtificial **C**hromosome) and BAC (**B**acterial **A**rtificial **C**hromosome) with repeat-containing oligonucleotides. The need to specifically identify and isolate STS-markers from these types of libraries is unquestionable. First, large-insert libraries are predominantly used in animal genetics, e.g. [3,4], as tools to identify candidate genes or to generate overlapping contigs of chromosomal regions that are associated with quantitative or economic trait loci (QTL or ETL). Secondly, the overall number of microsatellites present in a genome depends mainly on their complexity and size. Assuming a total size of 3 × 10^9^ bp and an estimated frequency of a dinucleotide repeat every 30–50 kb in mammals (as reviewed by [5]), a genome-wide figure of 100,000 microsatellite markers of that kind can be assumed [6]. However, only approximately 1,200 porcine microsatellites have been reported so far [7]. Furthermore, both the total number and the distribution of the loci are still not sufficient to have well-distributed microsatellite coverage throughout the genome or for several chromosomes, e.g. *SSC18 *[8]. The objective of the present study was the selective generation of microsatellites from PAC-clones, which were prior to STS development isolated from the porcine PAC library TAIGP714 [3] by a three-dimensional PCR screening strategy [9]. Eight of the eleven clones harbored functional or positional candidate genes involved in health, reproduction, production, and regulation, whereas the other three clones have been used in the attempt to construct a PAC contig covering *SSC16q11-13* (Table 1).

**Table 1:** Primers used for selecting the PAC clones from TAIGP714 large-insert library

PAC Clone	Gene/Marker	Forward Primer (5'-3') Reverse Primer (5'-3')	Size (bp)	References
TAIGP714M09100Q	GUSB	GTCTGTGTCTGACTTCTACACTCTC GCGGTCACAGGCTGCATCACCT	504	[24]
TAIGP714N07113Q	HEXB	GGAAGCTATTCTTTGTCTCATGT CTTTTCCCCAAGACCGTGAAT	132	[25]
TAIGP714I04060Q	CALCA	CCCTCACTCTTACCTCTAACC AGCTAAGCGGTGCAGTAATC	398	[26]
TAIGP714P05202Q	HoxA10	CAGCCAACTGGCTCACGGCA AGTTGGCTGTGAGCTCCCGG	239	[27]
TAIGP714O11196Q	HYAL3	GATTGGGAGGAGTGGTGTC GAGGTAGATGCTGGGGAAG	363	[28]
TAIGP714I22103Q	SPRMTK	GCGAGATGACATGACTGTCT CTGTACCCATGGCACGCACA	254	[29]
TAIGP714F10061Q	SW813	TCAGTTATTTCTGGCTATCATCTC TTGATGTAGACCACCCAGCTAGTG	98–114	[12]
TAIGP714L02061Q	S0111	AGTTGATTTAAAATGTTGTGCCA AATATTTCAAAAAAAGGAATGCG	150–178	[10]
TAIGP714I23038Q	SW742	AATTCTACTTCTGGGGAGAGGG CTTTTGGGAACATTTCTGCC	193–227	[11]
TAIGP714N18001Q	LAMR1P1	GTCGTAACTTAAAGGGAG ATTTGGAAGTCAAGGTTGG	128	unpublished
TAIGP714H02175Q	PGK1	CTTCCATCCCAAGCATC TTCCCTTCTTCCTCCAC	384	[30]

## Results and discussion

Fifteen of the seventeen microsatellites (Table 2) were developed with sequencing primers containing one selective nucleotide at the 3'-end: (CA)_8_T (*S0701*, *S0703*, and *S0767*), (CA)_8_A (*S0702*, *S0704*, and *S0710*), (CA)_8_G (*S0705*, *S0706*, *S0712*, and *S0766*), (AC)_8_C (*S0709*), (AC)_8_G (*S0707 *and *S0715*), (AC)_8_T (*S0708 *and *S0711*). Characterization of microsatellites *S0713 *and *S0714 *was only accomplished by an improved discrimination of the PAC clone sequences with sequencing primers further extended at the 3'-end with a second nucleotide [(CA)_8_AT for *S0713 *and (CA)_8_GC for *S0714*]. The second nucleotide became necessary because the respective clones TAIGP714L02061Q (for *S0713*) and TAIGP714I23038Q (for *S0714*) contained additional (CA)_8_A or (CA)_8_G primer binding regions or motifs. Contrary, a further extension with three nucleotides at the 3'-ends of the primers did not result in additional microsatellites in any of the PAC clones or was not required. Therefore, we conclude that repeat primers with two 3'-nucleotides next to the repeat motif are sufficient to detect and sequence all repeats potentially present on a large-insert library clone. The results of our isolation strategy also indicate that two sequencing reactions (the reverse sequencing primer was designed based on the obtained sequences) seem to be sufficient in most cases to gain sequence information of high quality to amplify microsatellites (Table 2). Usage of sequencing primers degenerated at the 3'-end proved, however, to be inadequate as no sequence information at all was achieved. Also, to avoid overlapping primary sequences, oligonucleotides that basically extend the dinucleotide repeat at the 3'-end – such as (CA)_8_C and (AC)_8_A – are not recommended. TOMMI proved to be an efficient and reliable isolation strategy. Besides new STS-markers, six previously described microsatellites were also detected. Three of these loci, microsatellites *S0111 *[10], *SW742 *[11], and *SW813 *[12], were initially used as probes for the isolation of clones TAIGP714L02061Q, TAIGP714I23038Q, and TAIGP714F10061Q. The other three already described microsatellite sequences reside on TAIGP714C09004Q [GenBank: AJ440949 (repeat location: 3172–3231) and GenBank: AJ440950 (repeat location: 15831–15860 and 16007–16038)]. They were not further considered in this study as they were not regarded as novel. Independently of our effort, two other groups [13,14] introduced similar sequencing approaches to generate microsatellites from large-insert libraries. There are, however, several differences between our approach and the ones of the other groups in terms of sequence generation and selective amplification of microsatellites. Here, contrary to Waldbieser and colleagues [14] – who used trinucleotide repeat containing primers for sequencing – both gene-specific primers are not 5'-tailed with extra nucleotide stretches to enable either product labeling or to promote alleged non-template adenylation. Fujishima-Kanaya's group [13] used larger repeat compounds contributing to the primer [(CA/GT)_(10) _instead of (CA/GT)_(8)_]. Secondly, the sequencing primers consisted generally of three selective nucleotides at the 3'-end adjacent to the repeat motif (e.g. CNA/GVG). There, the first of the three terminal nucleotides was always identical with the starting nucleotide of the dinucleotide repeat primer used. In addition, primers contained a degenerated base according to the International Union of Biochemistry (IUB) codes at the second position from or directly at the 3'-end. Thirdly, determination of the double-stranded primary DNA sequence stretch was achieved by four sequencing reactions using both a CA-repeat containing primer plus a GT-repeat containing primer heading in the opposite direction and two reverse primers were developed based on the obtained sequence. Finally, they always designed an additional primer pair for the specific amplification of the microsatellite. In contrast, we used the single reverse sequencing primer in combination with a newly developed sequence specific primer (*S0766 *and *S0767*) or designed a new primer pair to amplify the microsatellite (*S0701 *to *S0715*).

**Table 2:** Forward and reverse sequencing primer

		Single reverse sequencing primer	
		
Locus	Initial sequencing primer (5'-3')	Primer sequence (5'-3')	Primer location (GenBank)
S0701	(CA)_8_T	CCCAGGAGATTGAATATAG	223–241 (AY253989)
S0702	(CA)_8_A	AAAAGCACCCAAAAAAGCC	156–174 (AY253990)
S0703	(CA)_8_T	CTTATGGAGGTTCTCAGG	55–72 (AY253991)
S0704	(CA)_8_A	GAGTGTGGGATAGACTG	119–135 (AY253992)
S0705	(CA)_8_G	GAAGGGTAGGTTAAAGGG	252–269 (AY253993)
S0706	(CA)_8_G	GGGAAACAGAAAATGGGG	156–173 (AY253994)
S0707	(AC)_8_G	GTGAGCAAATAATTCAGTG	116–134 (AY253995)
S0708	(AC)_8_T	CACAATTACTGCTTCTCTC	77–95 (AY253996)
S0709	(AC)_8_C	GAGTGAGCACCATTCTAAG	117–135 (AY253997)
S0710	(CA)_8_A	GCTTCATCACCCTGTTC	61–77 (AY253998)
S0711	(AC)_8_T	CATTTTTCAGAGGGAAGAG	86–104 (AY253999)
S0712	(CA)_8_G	GACCCTGGCATAGTATC	254–270 (AY254000)
S0713	(CA)_8_AT	GAAGATACTGTTCTATGGATAG	1–22 (AY254001)
S0714	(CA)_8_GC	CGTGTAGGTTGAAGACAAG	123–141 (AY254002)
S0715	(AC)_8_G	GAGTTGTGTTTTATGGAGTTG	43–63 (AY254003)
S0766	(CA)_8_G	AGACCTCCTATTAGAGGTGGA	519–539 (AY731063)
S0767	(CA)_8_T	CTAGAATGGAAAACAATCTGA	367–387 (AY731064)

The observed number of alleles per locus (monomorphic locus *S0709 *is not included in this calculation) in the heterogeneous sampling was as low as 2 (*S0702*) and as high as 22 (*S0713*), leading to an average number of 9.94 alleles, N_E _ranged from 1.05 to 11.54 and both H_T _and PIC from 0.05 to 0.91 (Table 3).

**Table 3:** Characteristics of TOMMI-microsatellites

PAC	Locus	Chr^1)^	Primer pair sequence (5'-3')	T_a_	Size range	Alleles	N_E_	PIC	H_T_	Repeat motif	GenBank
TAIGP714M09100Q	S0701	3p16-p14	GCAGAGTGATTCAGTTATAC	60	366–372	3	1.89	0.38	0.47	i(GT)14i(AT)6	AY253989
			TCATCTTCCCTACCACC								
TAIGP714M09100Q	S0702	3p16-p14	TTTGGGGGGTTTGTTTTTG	57	346–348	2	1.18	0.14	0.16	(GT)9	AY253990
			AATATAATTGGTGGCTCGG								
TAIGP714N07113Q	S0703	2p13-p11	AACCCACTGAACAAGGC	58	239–259	7	3.37	0.70	0.70	i(GT)11(AT)9	AY253991
			GCAAGACAGATACTACAGG								
TAIGP714N07113Q	S0704	2p13-p11	AGCTATCATCAGGAAATGC	58	265–285	11	5.34	0.81	0.81	(GT)18	AY253992
			GTTCTGTCGATTTTCTACTG								
TAIGP714I04060Q	S0705	2q21-q22	CAGGGGGTTAAAGATCAG	59	292–326	9	1.99	0.47	0.50	(GT)13	AY253993
			GGGGCACATAAAAGGAAG								
TAIGP714P05202Q	S0706	18q23-q24	CTGGGTTGCTAAAGAGAC	56	211–215	3	1.05	0.05	0.05	(GT)6	AY253994
			CACCTGAAGGATGTGAG								
TAIGP714O11196Q	S0707	13q21	GGTAGGGCTTACTTAACTC	56	163–196	11	6.89	0.85	0.86	i(GT)19i(GC)7(GA)10	AY253995
			GAGAGGGATGAGAATCAG								
TAIGP714I22103Q	S0708	3q11-q12	GTTAGTTTCAGGCGTATAG	56	349–397	16	6.57	0.84	0.85	(GT)15i(AT)25(GT)14(AT)11	AY253996
			CTGTGGTATAGGTCGAAG								
TAIGP714F10061Q	S0709	16q11-13	TTTAAGACACAGACAGCAG	58	151	1	1	0	0	i(GT)9	AY253997
			CAGCATCTACATCCAGAC								
TAIGP714F10061Q	S0710	16q11-13	CTCAGCACCTTACAAACC	58	326–387	14	3.88	0.72	0.74	i(TAAA)7(GT)9	AY253998
			TCCCAAACCAATCCACAC								
TAIGP714F10061Q	S0711	16q11-13	CAGAATCTAGCCTCAGCGTC	58	201–209	8	3.06	0.66	0.67	(GT)6(G)10	AY253999
			CACTCCATCCCTCCCAAG								
TAIGP714L02061Q	S0712	16q11-13	TGGCATTGCTATGGCTG	57	251–310	14	5.57	0.82	0.82	(GT)12	AY254000
			CACAACCACCTACATATCATC								
TAIGP714L02061Q	S0713	16q11-13	CATAATGCCCTCCACATC	54	263–317	22	11.54	0.91	0.91	(GT)17	AY254001
			CCATATCATCCAGCAATTC								
TAIGP714I23038Q	S0714	16q11-13	TCTAGCTGTCGTGTAGG	55	199–207	8	3.75	0.70	0.73	(GT)7	AY254002
			GAGGGATTACTCTGAGTTAAG								
TAIGP714I23038Q	S0715	16q11-13	GCCCTCCAGGACAAAAC	58	208–242	14	7.16	0.86	0.86	(GT)10i(GC)7i(GT)14	AY254003
			GCTGTGACGTAGGTTGG								
TAIGP714N18001Q	S0766	6q27-28	GTGTAGATATGTGTCTGTACA	58	439–471	14	6.98	0.86	0.86	(GAAA)4(CA)6i(CA)16	AY731063
			AGACCTCCTATTAGAGGTGGA								
TAIGP714H02175Q	S0767	Xq12-q13	TGACCATGTCTTGTGGTAA	53	239–247	3	2.04	0.40	0.51	(CA)11	AY731064
			CTAGAATGGAAAACAATCTGA								

T_a _= Annealing temperature; N_E _= Effective allele number; PIC = Polymorphism Information Content; H_T _= Heterozygosity; i = Interrupted sequence.

1) Physical assignment: *S0701 *to *S0708*, and *S0767 *[24-30]; *S0709 *to *S0715*, and *S0766 *(physical localisation unpublished, but results are available through the website of the INRA-UMN porcine rodent hybrid IMpRH panel [17]

Due to their isolation from partial genomic libraries selected for small insert sizes most of the publicly available porcine microsatellites lie within DNA-fragments of about 80 to 200 bp. Their potential combination in multiplex assays – also considering different annealing temperatures and technical limitations of the automated sequencers (limited number of available fluorescent dyes) – is therefore hampered. Hence, an enhanced number of genotypes per run can only be achieved by the integration of STS-markers covering a larger allelic spectrum. Thus, we intended and focused on the development of large amplicons for microsatellites by utilizing as much sequence information as possible for primer design. Indeed, fourteen STS-markers had allele sizes of at least 200 bp and for five of the isolated microsatellites, sequence information proved to be good enough to amplify allele sizes of at least 300 bp (Table 3).

By the guided isolation of STS-markers *S0709 *to *S0715 *from three *SSC16q* derived PAC clones (relative position 0 cM to 9.3 cM [7]; 2.33 STS-markers per clone), the marker density in this chromosomal region was improved remarkably. An average of 1.55 new microsatellites was isolated from PAC clones harboring functional candidate genes (*S0701-S0708*; *S0766 *and *S0767*). Considering all used PAC clones and developed STS-markers, 1.55 microsatellites per clone were isolated. As the PAC clones had an average length of 80 kb (as shown by pulsed-field-gel electrophoresis) the frequency of dinucleotide repeats every (30 to) 50 kb [5] was more or less confirmed. TOMMI holds therefore the potential to identify existing STS-markers linked/adjacent to e.g. candidate genes on large-insert library clones. Thus, in combination with a genome scan, respective putative candidate genes could either be transformed to or excluded as positional candidate genes prior to their complete structural characterization including SNP detection. Linkage mapping results for *S0701*, *S0705*, *S0707*, *S0711*, *S0712*, *S0713*, *S0715*, and *S0766 *are presented in Table 4. A comparison of their mapping positions with QTL positions (Pig Quantitative Trait Loci (QTL) database [15] reveal that *S0705 *(64.22 cM), *S0707 *(43.19 cM), and *S0766 *(102.50 cM) reside on the respective chromosomes exactly at QTL locations (*S0705*: backfat between the last 3^th ^and 4^th ^rib; *S0707*: early growth rate and water holding capacity; *S0766*: backfat thickness at first rib and intra-muscular fat). The other STS-markers are located in QTL spans of ± 5 cM. This indicates their immediate potential to further dissect these respective QTL regions.

**Table 4:** MARC marker information and linkage mapping results

Marker	Forward Primer (5'-3')	Reverse Primer (5'-3')	Number of Alleles	Allele Size Range (bp)	Number of Meioses	Linkage Position (Chr: cM)
S0701	TGTTTCAGGTACACAGCAGAGTG	AACGCGGTTTTGACCTACAG	3	161–167	48	3:31.4
S0705	TGGTTCAGATTGCTGTGGAG	ATACCTGCAAACGCTGACCT	5	182–200	62	2:64.3
S0707	GGTAGGGCTTACTTAACTC	GAGAGGGATGAGAATCAG	4	165–195	80	13:42.5
S0711	TCTGTTGCTGGCCATGAGT	GTTCTGGCAACCCAGTCCT	2	116–119	63	16:5.9
S0712	TTTGCACTCTGCTTTTGAAGA	GACCTGCACAACCACCTACA	6	173–221	134	16:0.6
S0713	AGCATAATGCCCTCCACATC	GTGGCACCAACAGATGAATG	8	157–185	162	16:0.0
S0715	CCCTCCAGGACAAAACATTC	TTTGAGGGAAAGAGGTGGAG	6	191–213	114	16:9.3
S0766	TAGAAACCTGCCCATTGAGG	AGGCAGGGACAGGGTCTATT	7	122–144	122	6:102.6

## Conclusion

The sequencing strategy described in this study provides a targeted, inexpensive and fast method to develop microsatellites from large-insert libraries. It is also well suited to generate polymorphic markers for selected chromosomal regions and contigs of overlapping clones and yielded sufficient high quality sequence data to develop marker amplicons greater than 250 bases.

## Methods

### PAC clone isolation and physical mapping

Prior to STS development, a total of 11 clones were isolated from the porcine PAC library TAIGP714 [3] by a three-dimensional PCR screening strategy. PAC-DNA preparations were done according to the manufacturer's protocol (Qiagen, Hilden, Germany). The physical assignment of the PAC clones was performed by Fluorescence *in situ* Hybridization (FISH) as described in [16] or alternatively by analysis of the INRA-UMN porcine radiation hybrid (IMpRH) panel [17]. Microsatellite primers (Table 3) were used to RH map *S0703*, *S0704 *and *S0708 *– *S0715*. Marker assignment of *S0701*, *S0702*, *S0705 *– *S0707*, *S0766 *and *S0767 *was performed with primers from further sequence segments of the PAC clones.

### Microsatellite generation and characterization

**Figure 1:**
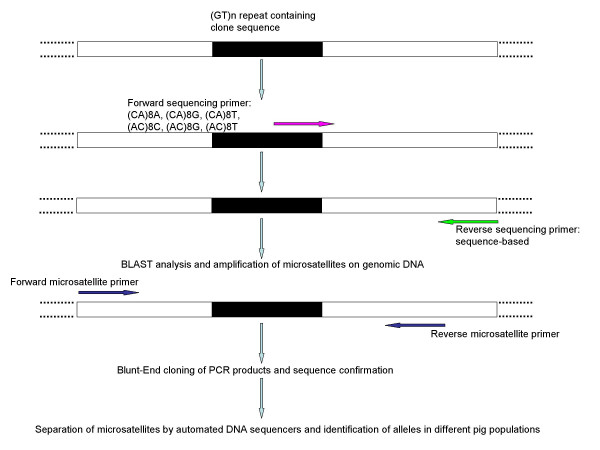
Generation of STS-markers by TOMMI.

All sequencing reactions and the separation of microsatellites were performed on an ABI PRISM^® ^3100 DNA analyzer (ABI, Weiterstadt, Germany). Sequencing reactions were done using the BigDye™ Terminator (v 3.0) Cycle Sequencing Kit (ABI, Weiterstadt, Germany). DNA sequencing was performed using 10 pmol of the respective oligonucleotide, 1 μl BigDye Premix and 50–100 ng of purified plasmid DNA as template in a total volume of 10 μl. Sequencing conditions were 96°C for 30 s followed by 30 cycles of 96°C for 10 s, the respective annealing temperature for 5 s and 60°C for 4 min. The optimal annealing temperature for the repeat containing primer was between 50°C and 52°C, except for the generation of sequences for *S0714*, which were at 56°C. To generate STS-markers, oligonucleotides containing repeat motifs (CA)_8 _respectively (AC)_8 _at the 5'-end and few (one or two) non-repetitive bases at the 3'-end were originally used as sequencing primers. Based on the obtained sequence, specific primers were developed and used as reverse oligonucleotides to determine the composition of the repeat region and its 5'-flanking region (Table 2; Figure 1). BLAST comparison followed sequence determination to verify the novelty and uniqueness of the obtained sequences. Depending on the quality of the sequenced stretch, primers were developed to amplify seventeen STS-markers (*S0701 *to *S0715*; *S0766 *and *S0767*; Table 3). To confirm the sequence identity of the respective microsatellites [GenBank: AY253989 to AY254003, AY731063, and AY731064] on genomic DNA, the resulting PCR products were subcloned into the polylinker of the pGEM^®^-T vector (Promega, Mannheim, Germany) and three independent clones each were bi-directionally sequenced using standard sequencing primers SP6 (5'-ATT TAG GTG ACA CTA TAG AA-3') and T7 (5'-TAA TAC GAC TCA CTA TAG GG-3').

Evaluation of microsatellites and size determination of alleles were done with appropriate ABI-softwares GENESCAN (3.7) and GENOTYPER (3.6) using GENESCAN™-500ROX™ as internal size standard. Oligonucleotides were designed with the Oligo Selection Program [18] and synthesized by MWG Biotech (Ebersberg, Germany). To characterize size range, number of alleles, polymorphism information content (PIC), average heterozygosity (H_T_) and effective allele number (N_E_) of the microsatellites, STS-markers were separately amplified. PCR assays were performed at 54°C for *S0706*, *S0708*, *S0712*, *S0713*, *S0714*, and *S0767*, at 56°C for *S0701*, *S0702*, *S0703*, *S0705*, *S0707 *and *S0715*, and at 58°C for *S0704*, *S0709*, *S0710*, *S0711*, and *S0766 *in a RoboCycler Gradient 96^® ^(Stratagene, LaJolla, USA) using PURE Taq Ready-To-Go PCR Beads^® ^(Amersham Biosciences, Freiburg, Germany), along with the respective oligonucleotides (one labeled at the 5'-end alternatively with fluorescent dyes FAM, JOE or NED) and 50 ng of genomic porcine DNA in a volume of 12.5 μl (the concentration of each dNTP is 100 μM in 10 mM Tris-HCl (pH 9.0 at room temperature), 50 mM KCl and 1.5 mM MgCl_2_). In total, 336 unrelated pigs representing nine European breeds (9 Angeln Saddleback, 18 Bunte Bentheimer, 9 German Edelschwein, 15 German Landrace, 30 Hampshire, 27 Göttingen Minipig, 31 Pietrain, 12 Swabian-Haellian Swine, and 7 European Wild Boar), and six Chinese breeds (30 Chinese Jiangquhai, 28 Chinese Luchuan, 30 Chinese Minpig, 30 Chinese Rongchang, 30 Chinese Tibetan, and 30 Chinese Yushanhei) were investigated. The standard PCR profile was as follows: pre-denaturation at 92°C for 2 min, followed by 35 cycles of 92°C for 30 s, the optimal annealing temperature for 30 s, and 72°C for 30 s. The final cycle had an extension at 72°C for 10 min. PIC, H_T _and N_E _were estimated based on algorithms as introduced by Botstein and colleagues [19], Nei [20], and Kimura and Crow [21].

### Linkage mapping of STS-markers on the USDA-MARC linkage map

Seven families of the MARC Swine Reference Population were genotyped as described [22]. Amplified DNA was radioactively labeled, separated by denaturing polyacrylamide gel electrophoresis and visualized with autoradiography. To ensure accurate sizing and discrimination of alleles, amplification primers were redesigned to yield smaller products for all markers except *S0706*, *S0707 *and *S0709*. *S0767 *was not tested in this population. Four markers were not informative in the MARC Swine Reference Population (*S0702*, *S0706*, *S0709 *and *S0714*) and four primer sets failed to produce reliable products (*S0703*, *S0704*, *S0708 *and *S0710*). Genotypes were determined and entered into the MARC Genome Database. Each marker was initially assigned to a chromosome based on TWOPOINT results of CRIMAP [23], then multipoint linkage analyses determined the final location of each marker. Genotypic data were evaluated with CHROMPIC and corrections made if necessary. The final position reported is based on the current MARC swine linkage map. Amplification primers for the eight successfully mapped markers are presented in Table 4.

## Authors' contributions

KFC conducted the lab work to isolate and characterize *S0701 *to *S0715 *and CK to isolate and characterize *S0766 *and *S0767*. CK shared manuscript preparation and editing with KFC, supervised KFC's Ph.D. thesis, evaluated microsatellite data, and organized and provided DNA of the European pig breeds. KBK optimized and conducted fragment analysis and was responsible for evaluation of microsatellite data. JR assisted KFC in the beginning of the project. LSH organized DNA of the Chinese pig breeds. GAR conducted linkage mapping of the markers and edited the manuscript. BB proposed the idea, supervised and commented on the project, was responsible for funding and manuscript editing, and acts as head of the research group in Göttingen.
